# Comparative Study of Ki-67 Labeling Index Quantification by Eye-rolling, Manual Count, and Digital Image Analysis; An Approach with Caution

**DOI:** 10.30699/IJP.2024.2008346.3150

**Published:** 2024-03-29

**Authors:** Aminder Singh, Vikram Narang, Ankita Soni, Kajal Angural, Saveena Jindal, Bhavna Garg, Harpreet Kaur

**Affiliations:** *Department of Pathology, Dayanand Medical College and Hospital, Ludhiana, Punjab, India*

**Keywords:** Eye-rolling estimation, Ki-67 labeling index, Digital image analysis, Manual count

## Abstract

**Background and Objective:**

An accurate Ki-67 labeling index assessment is critical for managing a few tumors, like breast carcinomas and neuroendocrine tumors. We aimed to determine the degree of agreement between digital image analysis (DIA) and eye-rolling assessment (EE) and DIA and manual count (MC) for Ki-67 LI scoring.

**Methods:**

A total of 120 cases (both tru-cut biopsies and resected specimens) were selected during the study period from the institutional database, wherein the Ki-67 labeling index was performed. The selected cases were divided into two groups, i.e., breast neoplasms and other neoplasms. The correlation between DIA and EE and DIA and MC for Ki-67 LI scoring was calculated in both groups.

**Results:**

A total of 113 cases were analyzed for Ki-67 LI by three different methods (EE, MC, and DIA); 7 cases were rejected due to poor image quality. Ki-67 LI scoring by DIA and EE was highly correlated in both study groups with a Spearman's rank correlation coefficient of 0.809 (*P*=0.01) and 0.904 (*P*=0.01), respectively. Correlation between DIA and MC methods was also found to be almost perfect in both study groups with a Spearman's rank correlation coefficient of 0.974 (*P*=0.01) and 0.955 (*P*=0.01), respectively.

**Conclusion:**

ImmunoRatio is a free web-based digital image analysis application that can be used for Ki-67 LI assessment with considerable reliability and reproducibility. Yet, it carries a few limitations and demands a careful approach and final confirmation by an expert.

## Introduction

Ki-67 labeling index (Ki-67 LI) is the most common tumor proliferation marker, which immuno-histochemically determines the cell growth fraction. It is used as a marker for classification, prognostication, and treatment planning of different malignancies. Ki-67 LI generally correlates with clinical stage, prognosis, and outcome. The diagnostic significance of the Ki-67 LI varies with the tumor type, and an accurate estimation of Ki-67 LI is critical for disease management in a few tumors, such as breast carcinoma and neuroendocrine tumors (NETs).Ki-67 LI assessment by traditional eye rolling method carries a high inter-observer and/or intra-observer variability, as well as limited reproducibility (1). The European Neuroendocrine Tumor Society (ENETS)/World Health Organization (WHO) proposed counting 500 to 2000 tumor cells on immunohistochemistry (IHC) to ensure an accurate Ki-67 LI scoring (2). However, it is not a commonly used method in daily practice. Eyeballing/eye-rolling estimation (EE) remains the most widely used technique, though carries a risk of poor reproducibility and inter-observer and intra-observer agreement. Various methods have been developed over the past few years to overcome EE's limitations, such as manual count (MC) and computer-based digital image analysis (DIA). Recently, computer-based quantitative IHC using digital software has emerged as a reliable and reproducible technique, yet the data related to its use in clinical practice is limited.

In this study, Ki-67 LI was performed using three methods, i.e., EE, MC, and DIA, for all the study cases. We aimed to determine the degree of agreement between DIA and EE and DIA and MC for Ki-67 LI scoring. In addition, we assessed the advantages and limitations of the use of DIA for Ki-67 LI scoring in clinical practice.

## Material and Methods

This was a retrospective study conducted from 1^st^ May 2018 to 1^st^ April 2019 at a tertiary care center. A total of 120 cases were selected from the institutional database wherein the Ki-67 labeling index was performed. Both tru-cut biopsies and resected specimens were included in the study. Clinicopathological data were retrieved from the institutional records. Ethical approval was obtained from the institutional ethical committee, and informed consent was obtained from all the study participants. 

For immunohistochemistry, formalin-fixed, paraffin-embedded tumor tissue was cut in 2-3 µm thick sections and mounted on Poly-L-Lysine coated slides. The IHC was performed on Ventana GX system BenchMark automated immunostainer using FLEX Monoclonal Mouse Anti-Human Ki-67 antibody (clone MIB-1, DAKO). External on-slide control tissue (lymph node) was also stained in all the study cases. Ki-67 LI was assessed as a percentage of total tumor cells.

Assessment of KI-67 LI: The quantification of the Ki-67 LI for every Ki-67 immunostained slide was performed using three different methods as described below.

1. Eye-rolling estimation (EE) under the Olympus CX43 microscope: All the slides were independently reviewed by three pathologists. The average of all three results was obtained for data analysis and the Ki-67 LI was calculated as the percentage of total tumor cells. For homogenous tumors, random areas were selected for evaluation. However, in heterogeneously stained tumors, areas of the highest Ki-67-positive tumor cell density (hot spot) were selected. 

2. Manual count (MC) of scanned images by Phillips slide scanner: The slides were scanned by Phillips slide scanner. The scanned images were then screened, and 3 to 5 hot spots were marked for manual counting. A total of 500 tumor cells were counted, excluding all the stromal and inflammatory cells. The result was obtained by calculating the average percentage of brown-stained nuclei over total brown and blue-stained nuclei.

3. Computer-based digital image analysis (DIA) by ImmunoRatio software: The scanned images of slides by Phillips slide scanner were screened and two different fields representing the areas of the highest Ki-67-positive tumor cell density (hot spot) were selected. The images were then uploaded on the digital image analysis software – ImmunoRatio and the scoring of Ki-67 LI were obtained by calculating the average of two hotspot areas. The DIA software provides the results as an average percentage of the brown staining pixel signals over total brown and blue staining pixel signals.

As the majority of the cases in our study were of breast, the selected cases were divided into two groups, i.e., breast neoplasms and other neoplasms, including neuroendocrine tumors, for comparison. The correlation between DIA and EE and DIA and MC for Ki-67 LI scoring was calculated in both groups.

Statistical analysis: SPSS statistics V.25.0 for Microsoft Windows (Chicago, USA) was used to analyze data. A probability value (p-value) of less than 0.05 was considered statistically significant. The correlation between DIA and EE and DIA and MC for Ki-67 LI scoring was evaluated by Spearman's rank correlation. Spearman's rank correlation coefficient (Spearman's ρ) of 0 corresponds to no association and the value of 1 corresponds to perfect association.

## Results

IHC for Ki-67 LI was performed on 120 cases in the study period (May 2018 to April 2019). However, 7 cases were rejected for analysis due to the poor quality of digitally scanned images. Thus, a total of 113 cases were analyzed for Ki-67 LI by three different methods (EE, MC, and DIA).

Of all, the majority of cases were epithelial neoplasms of the breast (both tru-cut and mastectomy) (n=57) comprising of the invasive duct and lobular carcinoma, followed by neuroendocrine tumors of the liver, lung, and pancreas. Uterine and ovarian epithelial tumors were comprised of 9 cases. Four cases were hepatocellular carcinomas. Hematolymphoid cases included 13 cases and 10 mesenchymal tumors were also included in the study and leiomyosarcoma of both uterine and extrauterine was most of them, along with 2 other lesions (total number =12) as described in Table 1.

NHL: Non-Hodgkin's lymphoma; NET: Neuroendocrine tumor; LMS: Leiomyosarcoma; LPS: Liposarcoma; HCC: Hepatocellular carcinoma; NEC: Neuroendocrine carcinoma; PNET: Primitive neuroectodermal tumor; FGT: Female genital tract; GIST: Gastrointestinal stromal tumor; DSRCT: Desmoplastic small round cell tumor; DFSP: Dermatofibrosarcoma protuberans.

**Fig. 1:**
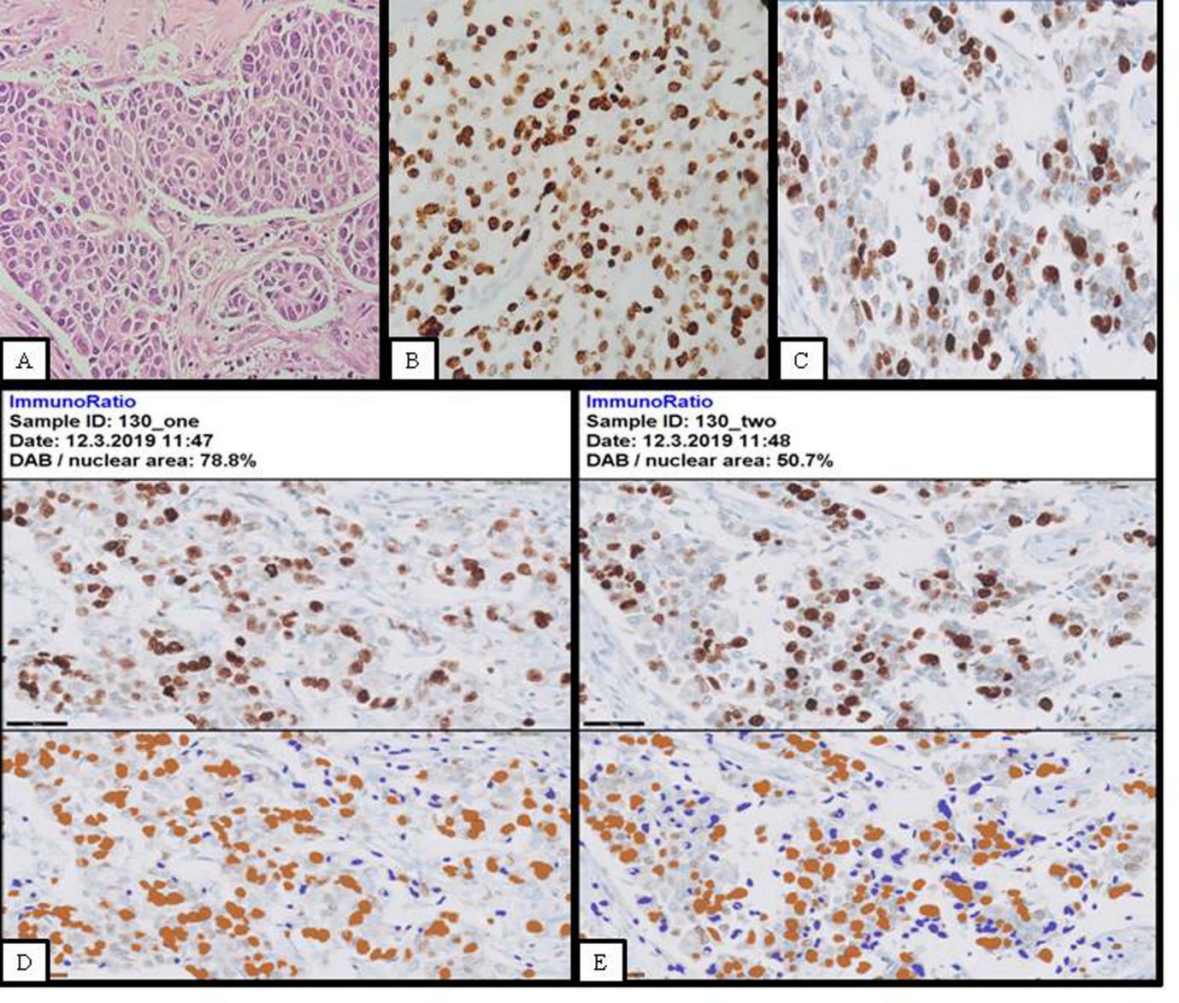
Photomicrograph (Hematoxylin and Eosin stain; x 100) showing histology of invasive breast carcinoma (Figure 1; panel A); Photomicrograph (Ki-67 immunostain; x 400) shows Ki-67 LI assessment by Eye-rolling estimation (EE) under the Olympus CX43 microscope (Figure 1; panel B); Photograph showing scanned image by Phillips slide scanner for Manual count (MC) (Figure 1; panel C); Photograph showing uploaded scanned images to ImmunoRatio software along with their pseudocolored images (two hotspot areas) (Figure 1; panel D and E)

A comparison of all three methods of Ki-67 LI assessment for a case of breast carcinoma (Figure 1).

**Fig. 2:**
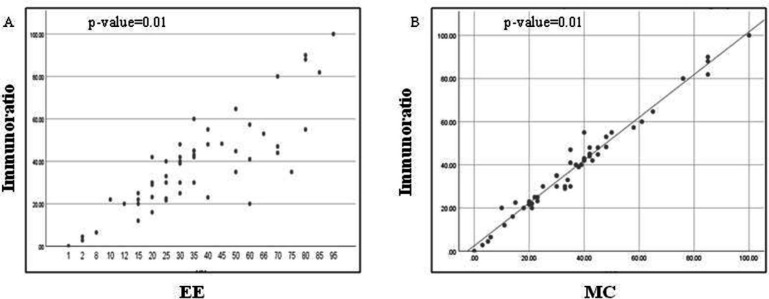
Comparison of the distribution of Ki-67 LI scores (in breast carcinoma cases) by EE and DIA by ImmunoRatio shows a significant correlation with Spearman's rank correlation coefficient of 0.809 (*P*=0.01) (Figure 2; panel A); by MC and DIA by ImmunoRatio shows Spearman's rank correlation coefficient of 0.974 (*P*=0.01) (Figure 2; panel B)

**Fig. 3:**
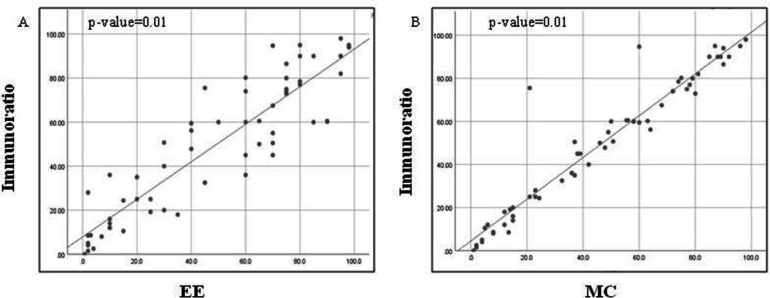
Comparison of the distribution of Ki-67 LI scores (in other neoplasms including neuroendocrine tumors) by EE and DIA by ImmunoRatio shows Spearman's rank correlation coefficient of 0.904 (*P*=0.01) (Figure 3; panel A); by MC and DIA by ImmunoRatio shows Spearman's rank correlation coefficient of 0.955 (*P*=0.01) (Figure 3; panel B)

Ki-67 LI scoring by DIA and EE was highly correlated in both the study groups with a Spearman's rank correlation coefficient of 0.809 (*P*=0.01) and 0.904 (*P*=0.01) respectively. Correlation between DIA and MC methods for Ki-67 LI assessment was also found to be almost perfect in both the study groups with a Spearman's rank correlation coefficient of 0.974 (*P*=0.01) and 0.955 (*P*=0.01), respectively (Table 2, Figures 2 and 3).

**Table 1:** Spectrum of all the cases included in the study period based on the tissue of origin

Epithelial	No. of cases	Mesenchymal	No. of cases	Hematolymphoid	No. of cases	Others (including neural crest tumors, round cell tumors and other lesions)	No. of cases
Breast cancer	57	LMS	06	NHL	11	PNET	03
NET	06	LPS	01	Hodgkin Lymphoma	01	Sex cord-stromal tumors	02
NEC	04	Schwannoma	01	Plasma cell neoplasm	01	GIST	02
FGT Cancer	09	Myxofibrosarcoma	01			Round cell tumor	01
HCC	04	DFSP	01			DSRCT	01
Metastatic deposits	02					Malignant melanoma	01
Poorly differentiated cancer	02					Pheochromocytoma	01
Adenocarcinoma	01					Pancreatic lesion	01
	85		10		13		12

**Table 2:** Comparison between EE, MC, and DIA for Ki-67 LI quantification

Type of cancer (n)	Methods of correlation	Spearman's rank correlation coefficient (Spearman's ρ)	P-value
Breast carcinoma (52)	DIA v/s EE	0.809	0.01
	DIA v/s MC	0.974	0.01
Other tumors including neuroendocrine tumors (61)	DIA v/s EE	0.904	0.01
	DIA v/s MC	0.955	0.01

## Discussion

The clinical significance of Ki-67 LI is well-established in a few neoplasms such as breast malignancies and neuroendocrine tumors. Its value in breast malignancies was established progressively over the last few years. High Ki-67 LI in breast cancer was recognized as an indicator of worse clinical outcomes, locoregional recurrence after modified radical mastectomy with negative lymph nodes, and pathological response after neoadjuvant chemotherapy (3). Molecular classification using multi-gene analysis or surrogate immunohistochemistry has a prognostic value that helps predict response to targeted therapies, degree of pathological response, and risk of relapse. Ki-67 LI is mandatory to differentiate breast carcinoma molecular subtypes luminal A and B to decide on additional chemotherapy. Ki-67 LI plays a pivotal role in neuroendocrine tumors as well and is a key element incorporated in the WHO classification of neuroendocrine tumors of all sites except the lung. The grading of NETs depends upon the critical cut-off values of mitotic count/Ki-67 LI that significantly affect the treatment plan and prognosis (4).

In this study, we performed Ki-67 LI in various neoplasms by all three quantification methods i.e., EE, MC, and DIA. The degree of agreement between DIA and EE and DIA and MC for Ki-67 LI scoring was evaluated. Ki-67 LI by DIA was found to be in almost perfect agreement with Ki-67 LI by EE and MC. However, the strength of correlation (Spearman's ρ) of DIA was better with MC than with EE in both the study groups. We did not correlate the study data with other clinicopathological parameters, as we considered that scanned images did not completely represent the individual cases. 

Among the three methods of Ki-67 LI scoring, the traditional method of Ki-67 LI estimation by eye-rolling carries a risk of poor inter/intraobserver agreement, yet can be used in breast carcinoma cases with a very low or very high Ki-67 LI. Ki-67 LI assessment by EE may not be satisfactory because of potential risks of overestimation/underestimation due to variable tumor cellularity and presence of the intratumoral and peritumoral inflammatory cells (5). Thus, in breast carcinoma cases with heterogeneous immunostaining and intermediate Ki-67 LI, MC, and DIA methods are preferred for correct molecular subtyping. Similarly, grading of neuroendocrine tumors is critical and demands an accurate estimation of Ki-67 LI, making the MC and DIA the preferred methods. In the MC method of Ki-67 LI estimation, every tumor cell is counted for its immunoreactivity to Ki-67 antibody on a scanned/printed digital image. Therefore, in the unavailability of DIA, MC is considered the "gold standard" method for Ki-67 LI scoring by the ENETS/WHO grading scheme for NETs (1). MC method was recommended by the International Ki-67 in Breast Cancer Working Group for breast carcinomas as well (6). However, counting every tumor cell individually makes this method very lengthy and tedious. To overcome these limitations, numerous digital image analysis (DIA) programs were developed.

The ImmunoRatio is one such web-based free DIA software to analyze immunostained slides which provide the results as an average percentage of the brown staining (DAB stained) pixel signals over total brown and blue staining (hematoxylin stained) pixel signals. ImmunoRatio software generates pseudocolored images which are displayed along with the original images for comparison. In cases of mismatch, the threshold for blue and brown signals may be adjusted for precise Ki-67 LI scoring. The literature review revealed four studies related to the assessment of Ki-67 LI by ImmunoRatio (7-10). In the present study, the Spearman's ρ between DIA and MC, in both the study groups was 0.974 (*P*=0.01) and 0.955 (*P*=0.01) respectively which was comparable to the study conducted by Yeo M-K* et al. *(Spearman's ρ=0.96, *P*=0.000) (10). However, Sundara Rajan* et al. *(7) and Fulawka* et al. *(9) reported a relatively lower degree of association with Spearman's ρ values of 0.87 and 0.83 respectively. 

To date, no global guidelines are available regarding the ideal method for assessment of Ki-67 LI. In this study, we did not find any discordance concerning the molecular subtyping of breast carcinoma by the three different methods. Yet, tumor grade changes from grade 1 to grade 2 in one of the cases of neuroendocrine tumors, after reanalyzing Ki-67 LI using DIA by ImmunoRatio. The role of DIA for Ki-67 LI scoring has been studied in other neoplasms as well (11-12). Yet, tumors other than breast carcinoma and NETs were not analyzed separately in the present study due to their insignificant numbers. 

We found DIA by ImmunoRatio as a reliable and less time-consuming alternative for Ki-67 LI scoring. However, results by ImmunoRatio might get distorted because of a few factors. First: ImmunoRatio software does not differentiate tumor nuclei from non-tumor nuclei (stromal cells and inflammatory cells). Though, this can be dealt with to a great extent by eliminating the non-tumor cells from analysis. Second: poorly fixed tissue causing heterogeneously stained nuclei may cause discordance of original and pseudo-colored images. In this study, seven cases were unsatisfactory for evaluation due to poor image quality.

## Conclusion

ImmunoRatio is a free web-based digital image analysis (DIA) application that can be used for Ki-67 LI assessment with considerable reliability and reproducibility. It is a less time-consuming alternative to the manual counting method as documented by an almost perfect Spearman's rank correlation coefficient in the present study. Yet, analysis by this application carries a few limitations and demands a careful approach along with final confirmation by an expert. Also, global standardization of the Ki-67 LI assessment method is required for better patient management.

## Funding

No funding.

## Conflict of Interest

The authors declared no conflict of interest.
